# Efficient Broad-Spectrum Cyanophage Function Module Mining

**DOI:** 10.3390/microorganisms12081578

**Published:** 2024-08-02

**Authors:** Yujing Guo, Xiaoxiao Dong, Huiying Li, Wei Lin, Lei Cao, Dengfeng Li, Yiming Zhang, Jin Jin, Yigang Tong, Zihe Liu

**Affiliations:** 1 Beijing Advanced Innovation Center for Soft Matter Science and Engineering, Beijing University of Chemical Technology, Beijing 100029, China; 2 College of Life Science and Technology, Beijing University of Chemical Technology, Beijing 100029, China; 3 Key Laboratory of Marine Biotechnology, School of Marine Sciences, Ningbo University, Ningbo 315211, China; lidengfeng@nbu.edu.cn

**Keywords:** cyanobacteria blooms, cyanophage, function module, gene function

## Abstract

Cyanobacterial harmful algal blooms (CyanoHABs) cause health and environmental effects worldwide. Cyanophage is a virus that exclusively infects cyanobacteria. Using cyanophages to control blooms is the latest biological control method. However, little research on the genomics of cyanophages and the presence of numerous proteins with unidentified functions in cyanophage genomes pose challenges for their practical application and comprehensive investigation. We selected the broad-spectrum and efficient cyanophage YongM for our study. On the one hand, through rational analysis, we analyze essential genes, establish the minimal cyanophage genome and single essential gene modules, and examine the impact of essential modules on growth. Additionally, we conducted ultraviolet mutagenesis on YongM to generate more efficient cyanophages’ critical modules through random mutagenesis. Then, we sequenced and analyzed the functionality of the mutational gene modules. These findings highlight several gene modules that contribute to a deeper understanding of the functional components within cyanophage genomes.

## 1. Introduction

In recent years, cyanobacteria blooms have become more frequent, causing significant harm to the global water environment and water security and posing a threat to human health [1,2,3]. Cyanophage is a virus that exclusively targets cyanobacteria. Using cyanophage to control blooms is an environmentally friendly and cost-effective method [4]. Surprisingly, the advancement of synthetic biology has opened up new possibilities for creating artificial cyanophages to manage blooms [5]. However, there are a lot of unknown functional proteins in the genome [6,7]. Nevertheless, there are numerous unknown functional proteins in their genomes, and detailed structural information on cyanophages is limited [8], hindering further research and engineering effort. Therefore, it is urgent to explore the functional modules of cyanophages to establish a foundation for their comprehensive utilization.

YongM, identified as a *Myoviridae* cyanophage (GenBankID: MT426122.1), possesses a linear double-stranded DNA of 65,429 bp, encoding 93 open reading frames [9]. Notably, YongM exhibits high efficiency and a wide range of activity. It has a broad spectrum and high efficiency, capable of lysing 18 species of cyanobacteria, infecting and lysing the host within 24 h [9]. However, among the 65 open reading frames encoded hypothetical proteins, while others are annotated based on predictive methods, limiting opportunities for further refinement and alteration.

At present, artificial cyanophages have been assembled but do not have any function. There have been examples of successful transformation of truncated cyanophages, but these instances do not involve truncation based on gene function [10,11]. While the mechanisms of cyanophage infection remain poorly understood, there is limited information on modifying virulent cyanophages. Consequently, employing rational mutagenesis methods is considered a practical strategy for further research and development in this area. Various modification methods have been proposed to enhance phage functionality and applications. These methods include random mutagenesis using physical and chemical techniques [12,13], as well as recombinant phage production through genetic engineering approaches [14,15]. Techniques like Bacteriophage Recombineering of Electroporated DNA (BRED) enable precise and effective mutagenesis of phage genomes, allowing for gene deletions, substitutions, replacements, and tagging [16,17]. The CRISPR-Cas system has emerged as a powerful tool for editing phage genomes and understanding phage-host interactions [18]. There are also studies focusing on phage genome reboot [19]. Additionally, methods such as Chemically Accelerated Viral Evolution (CAVE) have been developed to rapidly enhance desired phage characteristics, like thermal stability [20]. Notably, a modification method known as REEP (REcombination, Enrichment, and PCR screening) has shed light on the integration and maintenance of lysogenic life cycles in cyanophages, highlighting the importance of key genes like integrase and attachment site [21].

Cyanophage as a novel approach for algal bloom control presents certain challenges, including an unclear infection mechanism and gene function. While there have been studies on artificial cyanophage, the analysis of key genes and key gene modules remains attention. Comparative analysis of bioinformatics and mutagenesis screening are the most effective means to obtain key gene modules. Here, we obtain the essential genes for the functional modules of YongM through bioinformatics analysis, construct a minimum of artificial cyanophages and single module plasmids, test the impact of these synthetic cyanophages on the growth of cyanobacteria under salt stress, and analyze the functions of these functional modules. On the other hand, cyanophages with enhanced infectivity were identified through iterative ultraviolet mutagenesis. Selected cyanophages underwent genome sequencing to obtain mutated sequences, which were then compared with the original sequences to identify mutant modules. Subsequent analysis of these mutant modules elucidated the changes crucial for the cyanophage infection of cyanobacteria in a reverse manner. This research provides an important reference for the development and application of artificial cyanophages and also provides a foundational understanding for the strategic design and modification of cyanophages.

## 2. Material and Methods

### 2.1. Strains, Plasmids, and Culture Conditions

All strains and plasmids used in this study are listed in Supplementary Table S1. *E. coli* strains were used for the construction of plasmids and cultivated in Luria–Bertani (LB) medium with an appropriate antibiotic (Ampicillin: 100 μg/mL) at 37 °C. Yeast strains were cultivated in YPD (Yeast Extract Peptone Dextrose) medium at 30 °C. Yeast strains carrying plasmids were grown in synthetic complete (SC) medium with the corresponding defect type. The *Synechocystis* PCC6803 strain was grown in BG11 medium (cat. HB8793, Hopebio, Qingdao, China) with an appropriate antibiotic (Spectinomycin: 25 μg/mL) at 30 °C with a light density of 2000 lux. Host cyanobacteria *Nostoc*. FACHB-596 was obtained from a freshwater cyanobacteria culture bank at the Institute of Hydrobiology, Wuhan Academy of Sciences, China. Cyanophage YongM was isolated from water samples of the Dianchi Lake in Kunming, Yunnan. It was preserved in the Center for General Microbiology of Microbial Culture Collection Management Committee, Conservation number: CGMCC No.18383. *Nostoc*. FACHB-596 strains were cultured under normal conditions at 25 °C with a light intensity of 2000 lux and exposed to 12 h light-dark cycles, and grown in BG11 medium. According to the proportion of YongM:host = 1:10, infecting logarithmic host cyanobacteria leads to an extended YongM suspension.

### 2.2. Minimal Genome Assembly

All primers used in this study are listed in Supplementary Table S2. ORF fragments were obtained by PCR (2×Phanta Flash Master Mix Dye Plus, Vazyme, Nanjing, China). Two adjacent fragments with a 35 bp homologous arm overlapped by PCR, and the fragment with two ORFs overlapped with the adjacent fragment again. This process was repeated until 5 to 6 larger fragments were obtained and finally assembled into a plasmid using yeast assembly. The DNA fragments, containing all the essential genes, were obtained through PCR, and the final plasmid, which was able to be expressed in cyanobacteria, was assembled using a recombinant enzyme (ClonExpress Ultra One Step Cloning Kit, Vazyme, Nanjing, China).

### 2.3. In Vitro Assembly

DNA fragments with 25 bp homologous arms were assembled by recombinant enzymes (ClonExpress Ultra One Step Cloning Kit, Vazyme, Nanjing, China), then transformed into *E. coli* competent cells, and the single colony was verified by enzyme digestion.

### 2.4. Transformation of Cyanobacteria

All cyanobacteria transformations were accomplished by the three-parent conjugation transfer method. Inoculated *Synechocystis* PCC6803 three days in advance until OD_750_ = 0.3. Inoculated *E. coli* Helper, which carries pRL443 and pRL623 at a 1% rate, until OD_600_ = 0.5 after approximately 5 h, as well as the *E. coli* Donor containing the plasmid to be transformed. Take 5 mL *E. coli* Helper and 5 mL *E. coli* Donor at 5000 rpm for 5 min, wash them three times with LB medium without antibiotics, and then resuspend into 100 μL LB, respectively. A total of 100 μL *E. coli* Helper and 100 μL *E. coli* Donor were mixed evenly and incubated at 37 °C for 30 min. Take 10 mL *Synechocystis* PCC6803, 5000 rpm for 5 min, washed once with BG11 and then re-suspended to 200 μL BG11. All *E. coli* and cyanobacteria were mixed evenly and cultured under light for 1 h. The mixture was diluted and coated on a plate (BG11 + 5%LB) with 0.45 μm filter membrane and no resistance. After 24 h of culture, the filter was transferred to a BG11 plate with the same resistance as *E. coli* Donor.

### 2.5. Growth Curve

The plasmids containing the key modules are transformed into Synechocystis PCC6803. Three clones were selected from the plate and inoculated into 5 mL BG11 medium (cat. HB8793, Hopebio, Qingdao, China) with appropriate antibiotics, respectively. After the strain grew to a stable stage, cells were collected, washed with water once, and inoculated into 30 mL BG11 medium (cat. HB8793, Hopebio, Qingdao, China) with an appropriate antibiotic and 5% NaCl. The initial OD_750_ = 0.1. OD_750_ was measured every day for a week. Strains were cultured under normal conditions at 30 °C with a light density of 2000 lux.

### 2.6. Mutation Condition

Take the mixture after the infection. Centrifuge at 10,000 rpm for 10 min, supernatant 0.22 μm filter membrane was taken to obtain YongM suspension. According to the experimental results, when the UV energy is 20 mJ, the number of algal plaques growing on the plate is 10% of that in the control group. Take 1 mL YongM suspension was irradiated with 20 mJ ultraviolet light by a UV crosslinker (Evenpure, Beijing, China). Infect algal liquid according to the ratio of cyanophage: algal liquid = 1:10, wait until algal liquid yellowed, continuous mutagenesis for about 10 generations. After cyanophage spots were grown, larger cyanophage spots were dug up, and the mutation was verified by sequencing. Untreated YongM suspension as a control, and each treatment was repeated three times. The titer of each treatment group was determined by using the double-agar plate method.

### 2.7. Growth and Stability

Expanded culture YongM, the double plate method was used to determine the titer of YongM, tested the OD_750_ of the host, and infected according to the following proportions: multiple of infection (MOI) was 0.01, 0.1, and 1, respectively. After 25 min incubation in the light incubator, the supernatant was removed and centrifuged at 6000 rpm at 4 °C for 10 min. The supernatant was removed and re-suspended in 5 mL BG11 medium for 24 h. The titers of cyanophages were measured, and three were parallel in each group.

1 mL YongM suspension was prepared at different temperatures (0 °C, 30 °C, and 60 °C) incubated for 1 h, different pH ddH2O (pH 3, 7, 10) incubated for 1 h, and different metal ions (10 mM Ca^2+^, 10 mM Mg^2+^) incubated for 25 min, and untreated was used as the control group. The titers of cyanophages were measured, three in parallel in each group.

### 2.8. Genome Enrichment and Extraction

Take 30 mL of infected supernatant, centrifuge at 4 °C and 6000 rpm for 20 min, and filter the supernatant with a 0.22 μm filter membrane. Fresh 20% (*w*/*v*) and 40% (*w*/*v*) sucrose solutions were used for density gradient centrifugation at 4 °C and 35,000 g for 1 h, the supernatant was discarded, and the precipitation was re-suspended with 200 μL 0.01 M PBS. The YongM genome was extracted using omega E.Z.N.A.^®^ SE Viral DNA/RNA kit (cat. R6871, Omega, Norcross, GA, USA). To corroborate the mutations, lllumina MiSeq sequencing platform (San Diego, CA, USA) was utilized to obtain paired-end reads.

## 3. Results

### 3.1. YongM Essential Gene Analysis

There are a total of 93 open reading frames in YongM, among which 65 are hypothetical proteins. We first eliminated hypothetical proteins and compared and analyzed the remaining ORFs. By comparing with the cyanophage that is close to YongM, *Nosto.* phage A1 (GenBank: KU234533.1), *Anabaena* phage Elbi (GenBank: MZ078141.1), and *Nostoc* phage N1 (GenBank: KU234532.1), homologous genes of ORF12 and ORF26 are missing, they could be non-essential genes. For the rest of the ORFs, it is speculated that ORF5, ORF15, ORF16, ORF18, ORF19, ORF21, ORF41, ORF54, ORF63, ORF77, ORF80 may be unnecessary after reviewing the previously reported literature. As shown in Table 1, 15 possible essential genes of YongM were obtained.

### 3.2. De Novo Construct Minimum Artificial Cyanophage

**Figure 1:**
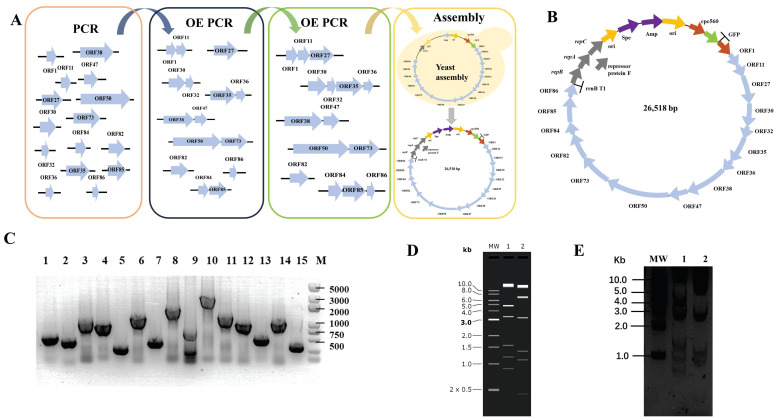
(**A**) Flow chart of de novo construct minimum YongM; (**B**) Design of functional module of minimum YongM; (**C**) PCR identification of single module plasmids, M: 5000 bp Maker; 1: ORF1, 645 bp; 2: ORF11, 507 bp; 3: ORF27, 1359 bp; 4: ORF30, 1098 bp; 5: ORF32, 327 bp; 6: ORF35, 1521 bp; 7: ORF36, 510 bp; 8: ORF38, 206; 7 bp; 9: ORF47, 885 bp; 10: ORF50, 3090 bp; 11: ORF73, 1572 bp; 12: ORF82, 1134 bp; 13: ORF84, 576 bp; 14: ORF85, 1176 bp; 15: ORF86, 348 bp; (**D**) Simulation of minimum YongM restriction agarose gel, MW: 1 kb DNA Ladder; 1: *SpeI*, 2: *NheI*; (**E**) Restriction of pLZ-GFP-YM agarose gel, MW: 1 kb DNA Ladder; 1: *SpeI*; 2: *NheI*. *SpeI* restriction: 14686 bp, 4857 bp, 3334 bp, 1560 bp, 1187 bp, 894 bp; *NheI* restriction: 13696 bp, 6712 bp, 3209 bp, 1351 bp, 1100 bp, 450 bp.

We designed the smallest artificial cyanophage from scratch using the essential gene sequences of YongM to investigate the most functional modules in the smallest cyanophage. We retain only the open reading frames of essential genes and eliminate meaningless sequences between the open reading frames. However, some open reading frames share common bases, such as the stop codon TAA and the start codon ATG of downstream genes. In such cases, an additional base A is inserted to maintain the integrity of each open reading frame without overlap (Figure 1A). The essential gene fragments were obtained by PCR, and the minimum YongM successfully assembled was obtained by OE PCR and yeast assembly. The yeast replication module was replaced by the cyanobacteria replication module, and the corresponding screening module was added. Finally, we obtained the minimum artificial cyanophage named pLZ-GFP-YM (Figure 1B). In addition, to verify the function of each module, we cloned every essential gene into a vector capable of replication and expression in cyanobacteria. This allowed us to confirm the impact of the essential gene on cyanobacteria. Surprisingly, ORF50 cannot be cloned, no matter what method is used. The results of plasmid construction are shown in Figure 1.

### 3.3. Effects of Salt Stress on Artificial Cyanophage

**Figure 2:**
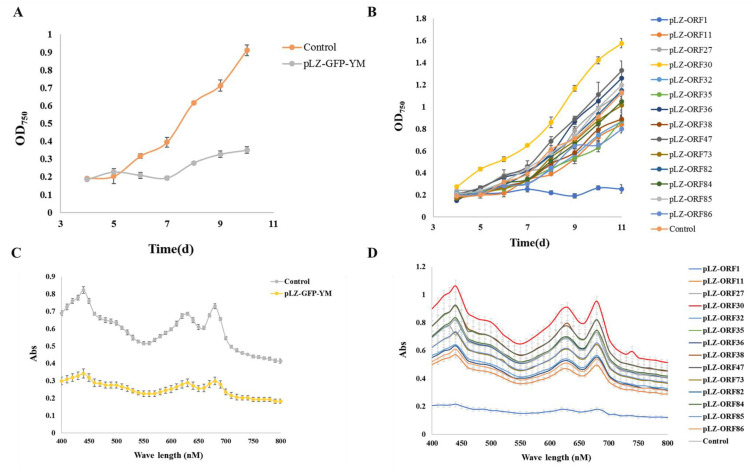
Growth curve and total absorption spectrum under 5% NaCl stress (**A**) Growth curve of minimum artificial cyanophage; (**B**) Growth curve of single gene modules; (**C**) Total absorption spectrum of minimum artificial cyanophage; (**D**) Total absorption spectrum of single gene modules.

**Figure 3:**
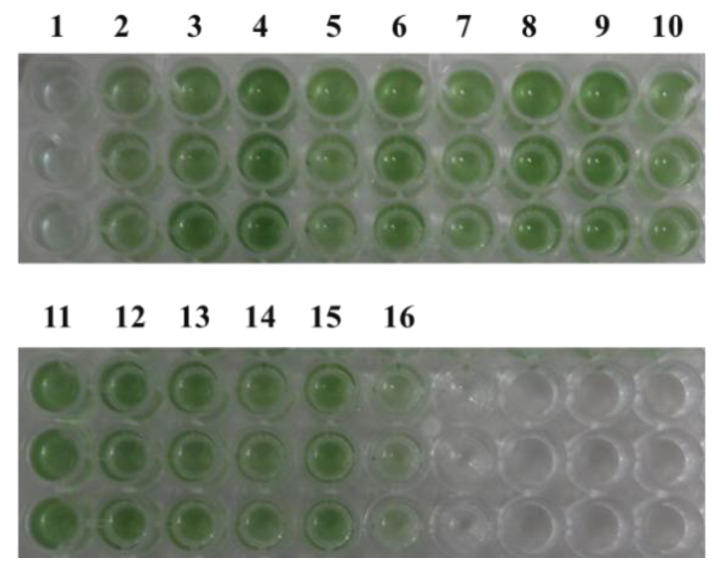
Strain color after 7 days of growth under 5% NaCl stress. 1: pLZ-ORF1; 2: pLZ-ORF11; 3: pLZ-ORF27; 4: pLZ-ORF30; 5: pLZ-ORF32; 6: pLZ-ORF35; 7: pLZ-ORF36; 8: pLZ-ORF38; 9: pLZ-ORF47; 10: pLZ-ORF73; 11: pLZ-ORF82; 12: pLZ-ORF84; 13: pLZ-ORF85; 14: pLZ-ORF86; 15: pLZ-Pcpc560-GFP (control); 16: pLZ-GFP-YM.

According to previous reports [10,11], under 5% NaCl stress, growth is regulated by an increase in osmotic pressure and inorganic ion concentrations [22,23]. Under typical circumstances, it is challenging to discern the impact of cyanophage modules on cyanobacteria, possibly due to the limited influence of individual modules under favorable growth conditions, with their effects becoming apparent only in extreme conditions. Therefore, the impact of minimum cyanophage and various single gene modules on the growth and pigment synthesis of the model alga *Synechocystis* PCC6803 6803 under 5% NaCl stress was investigated. The results are presented in Figure 2 and Figure 3. Compared with the control transferred to plasmid pLZ-Pcpc560-GFP, the minimum artificial cyanophage barely grew, indicating that the expression of certain genes on the minimum artificial cyanophage inhibited cell growth. The growth of most of the single gene-transferred strains was basically the same as the control, and the concentration of ORF30 was higher. However, the growth rate did not differ from that of the control, which may be related to the activity of the strain (Table S3). The results of full-wavelength scanning showed that the pigment level of the strain was essentially consistent with its growth. Interestingly, the strain with transferred ORF1 hardly grew and produced almost no synthetic pigments. The final OD_750_ after 7 days was even lower than that of the strain transferred to the minimum artificial cyanophage (Figure 3). This suggests that ORF1 may be an important gene for cyanophage infection and lysis of cyanobacteria. ORF1 is a thymidyate kinase [24] that appears to be associated with the recognition and adsorption of cyanobacteria [25].

### 3.4. Isolation and Stability Tests of Mutant Cyanophages

**Figure 4:**
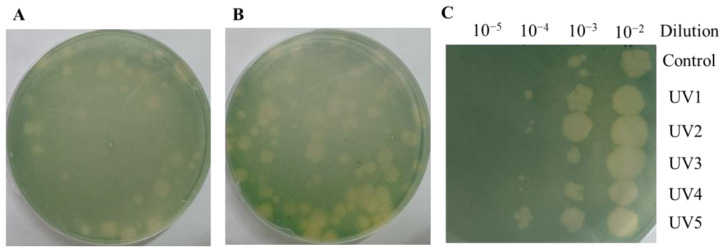
(**A**) Algal plaques after a single mutagenesis; (**B**) Algal plaques after 10 rounds of mutagenesis; (**C**) Infection of mutant cyanophages at the same concentration.

The above results only analyzed the ORFs that could be annotated. In order to explore more gene functions, we generated cyanophages with enhanced infection ability by mutagenesis and analyzed the mutated genes. As shown in Figure 4A, a single UV mutation could result in algal plaques of varying sizes. This demonstrates that the infection rate of mutant cyanophage YongM is not consistent, and cyanophages with a higher infection capacity can generate larger algal plaques. As shown in Figure 4B, when continuous UV mutagenesis was performed on cyanophages, the size of the resulting algal plaques varied greatly, and the number of large algal plaques was significantly higher than that from a single mutagenesis. The selected cyanophages were named UV1, UV2, UV3, UV4, and UV5. The infectivity of the five cyanophages is illustrated in Figure 4C. Using the same concentration of cyanophages, dilute the drop plates with different gradients and observe the size of the algal plaques. The algal plaques obtained through mutation are noticeably larger. Even after diluting the same gradient, UV-cyanophages can still generate algal plaques, demonstrating that the infection capability of mutated cyanophages has been enhanced.

**Figure 5:**
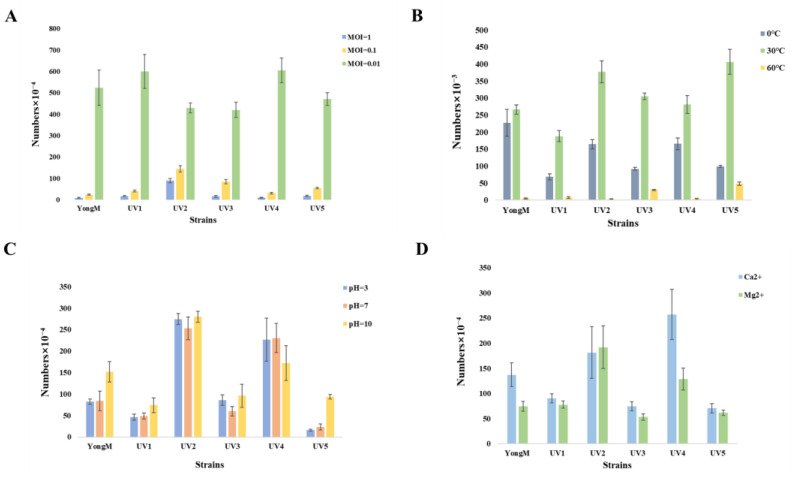
(**A**) MOI; (**B**) Thermal stability; (**C**) pH tolerance; (**D**) Metal ion sensitivity.

To assess the effectiveness of the mutant cyanophages, we conducted tests on their basic biological and physicochemical characteristics. The optimal infection number of cyanophages, different pH levels, various temperatures, and sensitivity to metal ions are presented in Figure 5. The optimal MOI for all cyanophages is 0.01. Thermal stability and pH tolerance experiments showed that the obtained cyanophages exhibited good thermal stability and pH tolerance, but they were inactivated at high temperatures (60 °C). After treatment with 10 mM Ca^2+^ and Mg^2+^ for 25 min, there is no effect on the activity of cyanophages. There was no significant difference between different cyanophages under different environmental conditions. These results are consistent with previous reports indicating that most cyanophages exhibit good stability [6].

### 3.5. Analysis of Mutation Site Distribution in Mutant Genome

**Figure 6:**
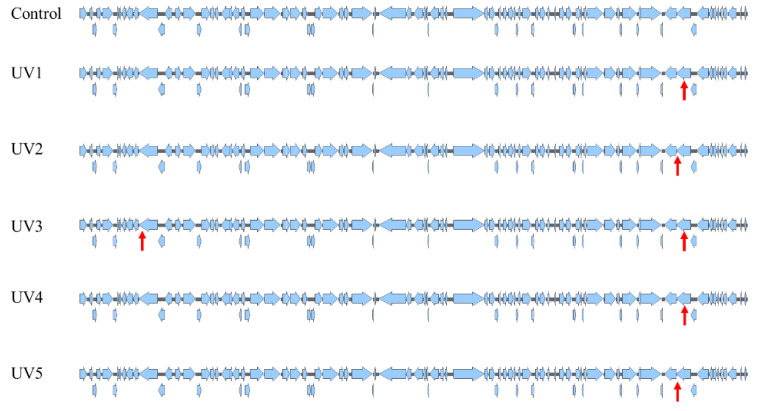
Base mutation information of mutated cyanophage. The red arrow indicates the mutation location.

Through sequencing, we obtained the genome sequence information of the mutant cyanophage. As shown in Figure 6 and Table 2, the five mutant cyanophage strains each produced a different mutation. Contrary to expectations, the number of these mutations was far fewer than expected. UV1 produced one non-synonymous mutation at 59,382 bp C mutated into T (3′ to 5′), resulting in the mutation of the predicted phage-related tail fiber protein from A to V with 195 amino acids. UV2 also generated one non-synonymous mutation at 58,773 bp, the G mutated into A (3′ to 5′), leading to the mutation of the predicted phage-related tail fiber protein from G to E with 398 amino acids. UV3 caused two non-synonymous mutations, one identical to UV1, and the other involved the removal of an A at 6196 bp, resulting in a mutation of the predicted alkaline phosphatase D protein. UV4 produced one non-synonymous mutation, the same as at UV1. UV5 produced one non-synonymous mutation, the same as in UV2.

The growth and other tests indicate that the infection capacity of mutant cyanophages has been enhanced to some extent, but the degree of improvement is not substantial. On one hand, the efficiency of traditional ultraviolet mutagenesis may not be high [26], and it may not be able to generate large-scale mutations and deletions [27]. On the other hand, the infection efficiency of the original cyanophage selected in this study may be high enough [9]. It is difficult to achieve further improvement because the growth rate of cyanophage is much lower than that of phage. Only 10 rounds of mutagenesis are insufficient to generate enough mutations [28]. The replacement of favorable genes is slow, and mutations with stronger infection ability are challenging to select quickly [20,29]. At the same time, no additional growth pressure from cyanophage is added during the mutation process [30]. As a result, there may be fewer mutations in the genome than expected. Furthermore, most of the mutations were associated with ORF83, and repeated mutations appeared, which may be because during the mutation process, mutations brought by this site can cause changes in the infection efficiency of cyanophage, which further proves the important role of this functional module.

## 4. Conclusions and Discussions

Cyanophage is a novel and safe method to control cyanobacteria blooms [1,31]. However, the efficient broad-spectrum cyanophage YongM among 70% are hypothetical proteins [32]. Here, we built a minimal cyanophage and identified a potentially crucial functional module through growth and pigment content measurements. Moreover, we mutated, sequenced, and analyzed to obtain YongM mutant modules, which provide important information for functional modules of the YongM genome. In conclusion, we identified significant functional modules in the cyanophage genome during the redesign and mutagenesis. We anticipate that these findings can serve as valuable references for future applications of artificial cyanophages.

The individual gene modules changed the growth and infection ability, suggesting that these genes may play a significant role in the infection process. The thymidylate kinase ORF1 may play a crucial role in the adsorption and infection of cyanobacteria [24,25]. Therefore, we hypothesize that ORF1 is a crucial gene of YongM in infection. Interestingly, no matter how the ORF50 expression system was constructed, it could not be cloned correctly. This suggests that ORF50 may be an essential gene for cells lysis. It has also been shown that some genes cannot be cloned into expression vectors and are toxic to *E. coli* [33]. Among the five strains of UV-cyanophage, all the cyanophages had mutations in ORF83. ORF83 is predicted to be a phage-related tail fiber protein. Fiber protein is a crucial component in the process of cyanobacteria cleavage. It aids cyanophages in binding to the cell surface, leading to subsequent tail contraction and DNA ejection [34]. The mutation of fiber protein may improve the adsorption efficiency and enhance the infection ability of cyanophage.

Unfortunately, artificial cyanophage is fraught with difficulties and challenges [31]. Although only a few mutant modules have been obtained, it is of great significance for us to further elucidate the infection mechanism of cyanophage. The next step involves conducting a comprehensive analysis of the acquired function modules, investigating their functionality, and elucidating their mechanism. Although cyanophages are an environmentally friendly means of bloom control, they may still have potential impacts on the original ecosystem. Cyanophages can lead to a significant reduction in cyanobacteria populations, release algal toxins and small molecular compounds, break the original balance of C, N, and P, cause the death or mutation of other aquatic organisms, and destroy the original water ecosystem. In addition, the cyanophage has the risk of escaping, which may infect other aquatic organisms, or horizontal gene transfer may occur. Therefore, it is very necessary to construct controllable artificial cyanophage to prevent cyanophage from escaping and ensure biosafety. Moreover, a storage technology for artificial cyanophages in a simulated natural environment needs to be developed to assess their effectiveness in preventing escape and ensuring safety in the management of water blooms.

## Figures and Tables

**Table 1:** Analysis of essential genes for YongM.

ORF	Start Site	End Site	Length	Predictive Function
ORF1	4603	5247	645	Thymidyate kinase
ORF11	6301	6807	507	capsid fiber protein
ORF27	13,086	14,444	1359	Large subunit terminase
ORF30	14,445	15,542	1098	the major capsid protein
ORF32	15,543	15,869	327	putative tail-component
ORF35	15,870	17,390	1521	Tail connector protein
ORF36	17,391	17,900	510	Tail connector protein
ORF38	17,901	19,967	2067	Tail-Associated Protein
ORF47	22,671	23,555	885	DNA polymerase III subunit alpha
ORF50	23,556	26,645	3090	primase
ORF73	27,510	29,081	1572	DNA translocase FTSK
ORF82	32,499	33,632	1134	Phage Tail Collar Domain
ORF84	33,633	34,208	576	Phage tail protein
ORF85	34,209	35,383	1176	Baseplate wedge protein gp7
ORF86	35,384	35,731	348	lysozyme

**Table 2:** Mutant Gene information.

Strain	ORF	Mutation Site (bp)	Codon Mutate	AA Mutate	Predictive Function
UV1	ORF83	59,382	GCT <-> GTT	A <-> V	Phage-related tail fiber protein
UV2	ORF83	58,773	GGA <-> GAA	G <-> E	Phage-related tail fiber protein
UV3	ORF12	6196	Delete A		Alkaline phosphatase D
UV3	ORF83	59,382	GCT <-> GTT	A <-> V	Phage-related tail fiber protein
UV3	ORF90	63,358	Synonymous mutation		
UV4	ORF83	59,382	GCT <-> GTT	A <-> V	Phage-related tail fiber protein
UV5	ORF83	58,773	GGA <-> GAA	G <-> E	Phage-related tail fiber protein

## Data Availability

The original contributions presented in the study are included in the article/Supplementary Material, further inquiries can be directed to the corresponding authors.

## Supplementary Materials

The following supporting information can be downloaded at: https://www.mdpi.com/article/10.3390/microorganisms12081578/s1, Table S1: strains and plasmids; Table S2: primers; Table S3: Specific growth rate of cyanobacteria transformed different gene modules.
