# Supplementary material for: Efficient Broad-Spectrum Cyanophage Function Module Mining
Source: Microorganisms. 2024 Aug 2;12(8):1578. doi: 10.3390/microorganisms12081578 (PMC11356776; doi:10.3390/microorganisms12081578)
Supplement: Supplementary file 1 [file microorganisms-12-01578-s001.zip › microorganisms-3104529-supplementary.pdf]

Supplementary

Efficient broad-spectrum cyanophage function module mining

Yujing Guo [a] †, Xiaoxiao Dong [b] †, Huiying Li [a], Wei Lin [b], Lei Cao [b], Dengfeng Li [c], Jin Jin [a] *, Yigang Tong [a, b] *, Zihe Liu [a, b, *]

[a] Beijing Advanced Innovation Center for Soft Matter Science and Engineering, Beijing University of Chemical Technology, Beijing, China

[b] College of Life Science and Technology, Beijing University of Chemical Technology, Beijing, China

c Key Laboratory of Marine Biotechnology, School of Marine Sciences, Ningbo University, Ningbo, 315211, China

† These authors contributed equally to this work.

* These authors are co-senior authors (email: zihe@mail.buct.edu.cn)

Supplementary Table S1 strains and plasmids

strains		Sources
E. coli DH5α	*supE44* Δ(*lacZYAargF*)*U169*(Φ*80lacZ* Δ*M15) hsdR17 recA endA1 gyrA96 thi-1 relA1*	Lab Store
S. cerevisiae BY4741	MATa *his3Δ1 leu2Δ0 met15Δ0 ura3-52*	Lab Store
Synechocystis PCC6803		Gift from Dr. Sun
YongM	Wild type cyanophage	Gift from Prof. Jiang
plasmids		
pSC-596-GFP	Amp, Spe, *CEN/ARS, LEU,* P_{cpc560}, P_{TEF1}, GFP, *repABC, ori*	This study
pLZ-GFP	Amp, Spe, *CEN/ARS,* P_{cpc560}, GFP, *repABC, mobABC, ori*	This study
miniYongM	Amp, *CEN/ARS, LEU* P_{cpc560}, YongM essential gene, rrnBT1	This study
miniYongMGFP	Amp, *CEN/ARS, LEU* P_{cpc560}, YongM essential gene, GFP, rrnBT1	This study
pLZ-YongM-GFP	Amp, Spe, P_{cpc560}, YongM essential gene, GFP, rrnBT1	This study
pLZ-ORF1	Amp, Spe, *CEN/ARS,* P_{cpc560}, YongM orf1, rrnBT1, *repABC, mobABC, ori*	This study
pLZ-ORF11	Amp, Spe, *CEN/ARS,* P_{cpc560}, YongM orf11, rrnBT1, *repABC, mobABC, ori*	This study
pLZ-ORF27	Amp, Spe, *CEN/ARS,* P_{cpc560}, YongM orf27, rrnBT1, *repABC, mobABC, ori*	This study
pLZ-ORF30	Amp, Spe, *CEN/ARS,* P_{cpc560}, YongM orf30, rrnBT1, *repABC, mobABC, ori*	This study
pLZ-ORF32	Amp, Spe, *CEN/ARS,* P_{cpc560}, YongM orf32, rrnBT1, *repABC, mobABC, ori*	This study
pLZ-ORF35	Amp, Spe, *CEN/ARS,* P_{cpc560}, YongM orf35, rrnBT1, *repABC, mobABC, ori*	This study
pLZ-ORF36	Amp, Spe, *CEN/ARS,* P_{cpc560}, YongM orf36, rrnBT1, *repABC, mobABC, ori*	This study
pLZ-ORF38	Amp, Spe, *CEN/ARS,* P_{cpc560}, YongM orf38, rrnBT1, *repABC, mobABC, ori*	This study
pLZ-ORF47	Amp, Spe, *CEN/ARS,* P_{cpc560}, YongM orf47, rrnBT1, *repABC, mobABC, ori*	This study
pLZ-ORF73	Amp, Spe, *CEN/ARS,* P_{cpc560}, YongM orf73, rrnBT1, *repABC, mobABC, ori*	This study
pLZ-ORF82	Amp, Spe, *CEN/ARS,* P_{cpc560}, YongM orf82, rrnBT1, *repABC, mobABC, ori*	This study
pLZ-ORF84	Amp, Spe, *CEN/ARS,* P_{cpc560}, YongM orf84, rrnBT1, *repABC, mobABC, ori*	This study
pLZ-ORF85	Amp, Spe, *CEN/ARS,* P_{cpc560}, YongM orf85, rrnBT1,	This study

	repABC, mobABC, ori	
pLZ-ORF86	Amp, Spe, *CEN/ARS,* P_{cpc560}, YongM orf86, rrnBT1, *repABC, mobABC, ori*	This study

Supplementary Table S2 primers

The lowercase part represents the homologous arm, and the underlined part represents the cleavage site and N20.

Name	Sequence
gfpF	ATGTCTAAAGGTGAAGAATTATTCA
oriF	CAATTGAGTTCTTTTACCCTCAGCC
oriR	TCAGCCTGCCGCCTTGGGCCGGGTG
pcpc560R	TGAATTAATCTCCTACTTGACTTTA
repAF	ATGGCTACCCATAAGCCTATCAATA
repBR	TGATTGCCTCCTTTGCAGGCAGTTG
tcyc1R	GCAAATTAAAGCCTTCGAGCGTCCC
tef1F	CCACACACCACGGCTCTAAAGTGCT
HA F	aaaattttgaaattgatatcaacaatcccatttaaCAAATAAAACGAAAGGCTCA
HAGFP R	gagtaaggtttttcgacattatatatagcgttcatTTATAGCTTGGATTTGTACA
pp1 R	ccgtttaaaatgccgataaatttgtttgacgacatTTAGCATAAATTTAAAGTTT
pp11 F	aaaagtggcttgacaaaactttaaatttatgctaaATGTCGTCAAACAAATTTAT
pp11 R	aggatgataactatcaacaataggaaagctagccatTACGCAATGCTTATAGCGT
pp27 F	actttgaatacaacgacgctataagcattgcgtaATGGCTAGCTTTCCTATTGTT
pp27 R	aatagttcagtaggtgttaaattcgttaaaggcattTAGTTTTGGTCAGCGTCAA
pp30 F	tatcttatagtccgtttgacgctgaccaaaactaaATGCCTTTAACGAATTTAAC
pp30 R	tcattgaataagtaaccgcttttagaaagtatcatTTACTGTGTAATGTCTCTCA
pp32 F	attctattagtgtaatgagagacattacacagtaaATGATACTTTCTAAAAGCGG
pp32 R	gtcccaatattaacgccatttaaaaagttagtcatTTAACTGCCTAGATTGTATA
pp35 F	ttatagaaagtgttatatacaatctaggcagttaaATGACTAACTTTTTAAATGG
pp35 R	ctatttatagaaaacggtcttttagaaactgccattTATACGACTGGCGCGGTTC
pp36 F	gtacagcgacagaaggaaccgcgccagtcgtataaATGGCAGTTTCTAAAAGACC
pp36 R	aaatctactgaaataacgtgatttctattagacatTTACAATCCTGTTTCAGCTA
pp38 F	aaccggccggtcaagtagctgaaacaggattgtaaATGTCTAATAGAAATCACGT
pp38 R	atcgcttcattgtactttgcaatgttggttatcatTTACGCATACTCATTTAATG
pp47 F	ctcaatggcaaggaacattaaatgagtatgcgtaaATGATAACCAACATTGCAAA
pp47 R	ccggacaattttttttggtcatttgttgtagacatCTATTCTATCACAATTTTGC
pp50 F	gctcggaaatacttggcaaaattgtgatagaatagATGTCTACAACAAATGACCA
pp50 R	tcagactttaaaaagttgcctagtggtttcttcatTCACATATCGATATATTGAT
pp73 F	ttgtgaatcgtgccgatcaatatatcgatatgtgaATGAAGAAACCACTAGGCAA
pp73 R	aggatatcaccattgttaaaaacttttctaggcatTCATTCTAAATTGCTAAAAA
pp82 F	atttgattgaaaagctttttagcaatttagaatgaATGCCTAGAAAAGTTTTTAA
pp82 R	tctatattccaagccgtgttaccatctttactcatTTACACTTTCACAAAAACTC
pp84 F	gctttttagtttttagagtttttgtgaaagtgtaaATGAGTAAAGATGGTAACAC
pp84 R	actaattgaggtgcgtttaagtctgttactgtcatCTATAAATCGTCGTATCTGA
pp85 F	ggtgtaaatatcaagtcagatacgacgatttatagATGACAGTAACAGACTTAAA
pp85 R	ccatttattattctaggggataaacatataacatTTATTCCTCAGGGTTTGTGA
pp86 F	ataatataataaccatcacaaaccctgaggaataaATGTTATATGTTTATCCCCC
pp86 R	gcccagtctttcgactgagcctttcgttttatttgttaaATGGGATTGTTGATAT

vectorF	CAAATAAAACGAAAGGCTCA
vectorR	TGAATTAATCTCCTACTTGA
orf1F	tcaagtaggagattaattcaATGAACGCTATATATAATGT
orf1R	tgagcctttcgttttatttgTTAGCATAAATTTAAAGTTT
orf11F	tcaagtaggagattaattcaATGTCGTCAAACAAATTTAT
orf11R	tgagcctttcgttttatttgTTACGCAATGCTTATAGCGT
orf27F	tcaagtaggagattaattcaTGGCTAGCTTTCCTATTGTT
orf27R	tgagcctttcgttttatttgTTAGTTTTGGTCAGCGTCAA
orf30F	tcaagtaggagattaattcaATGCCTTTAACGAATTTAAC
orf30R	tgagcctttcgttttatttgTTACTGTGTAATGTCTCTCA
orf32F	tcaagtaggagattaattcaATGATACTTTCTAAAAGCGG
orf32R	tgagcctttcgttttatttgTTAACTGCCTAGATTGTATA
orf35F	tcaagtaggagattaattcaATGACTAACTTTTTAAATGG
orf35R	tgagcctttcgttttatttgTTATACGACTGGCGCGGTTC
orf36F	tcaagtaggagattaattcaATGGCAGTTTCTAAAAGACC
orf36R	tgagcctttcgttttatttgTTACAATCCTGTTTCAGCTA
orf38F	tcaagtaggagattaattcaATGTCTAATAGAAATCACGT
orf38R	tgagcctttcgttttatttgTTACGCATACTCATTTAATG
orf47F	tcaagtaggagattaattcaATGATAACCAACATTGCAAA
orf47R	tgagcctttcgttttatttgCTATTCTATCACAATTTTGC
orf50F	tcaagtaggagattaattcaATGTCTACAACAAATGACCA
orf50R	tgagcctttcgttttatttgTCACATATCGATATATTGAT
orf73F	tcaagtaggagattaattcaATGAAGAAACCACTAGGCAA
orf73R	tgagcctttcgttttatttgTCATTCTAAATTGCTAAAAA
orf82F	tcaagtaggagattaattcaATGCCTAGAAAAGTTTTTAA
orf82R	tgagcctttcgttttatttgTTACACTTTCACAAAAACTC
orf84F	tcaagtaggagattaattcaATGAGTAAAGATGGTAACAC
orf84R	tgagcctttcgttttatttgCTATAAATCGTCGTATCTGA
orf86F	tcaagtaggagattaattcaATGTTATATGTTTATCCCCC
orf86R	tgagcctttcgttttatttgTTAAATGGGATTGTTGATAT
orf85F	tcaagtaggagattaattcaATGACAGTAACAGACTTAAA
orf85R	tgagcctttcgttttatttgTTATTCCTCAGGGTTTGTGA

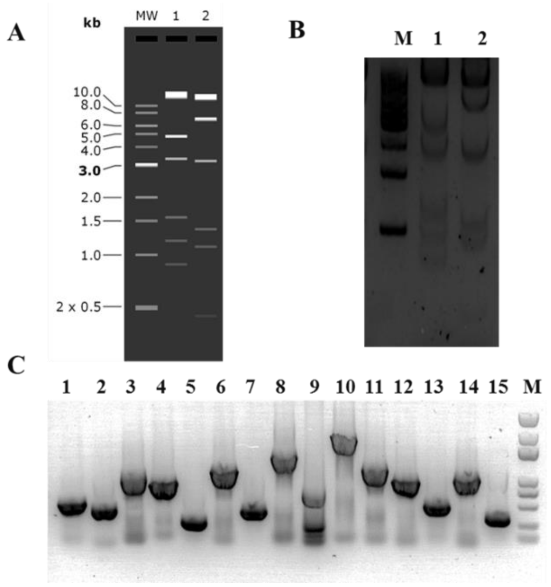

Supplementary Figure 2 Restriction of minimum YongM

A: Simulated agarose gel, MW: 1 kb DNA Ladder; 1: pLZ-GFP-YM, *Spe*I, 14686 bp, 4857 bp, 3334 bp, 1560 bp, 1187 bp, 894 bp; 2: pLZ-GFP-YM, *Nhe*I, 13696 bp, 6712 bp, 3209 bp, 1351 bp, 1100 bp, 450 bp;

B: M: 1 kb DNA Ladder; 1: pLZ-GFP-YM, *Spe*I; 2: pLZ-GFP-YM, *Nhe*I;

C: PCR identification, M: 5000 bp Maker; 1: ORF1, 645 bp; 2: ORF11, 507 bp; 3: ORF27, 1359 bp; 4: ORF30, 1098 bp; 5: ORF32, 327 bp; 6: ORF35, 1521 bp; 7: ORF36, 510 bp; 8: ORF38, 206; 7 bp; 9: ORF47, 885 bp; 10: ORF50, 3090 bp; 11: ORF73, 1572 bp; 12: ORF82, 1134 bp; 13: ORF84, 576 bp; 14: ORF85, 1176 bp; 15: ORF86, 348 bp
